# Relationship between TYG mediated pregnancy diabetes history and SII in American women: a retrospective cohort study of NHANES 2011-2018

**DOI:** 10.1186/s12902-025-02042-8

**Published:** 2025-09-22

**Authors:** Yanan Duan, Ru Zhang, Yan Zhang, Yuanxuan Ma, Miaomiao Li, Wenke Zhang, Shiguo Liu

**Affiliations:** 1 Department of Gynecology, Affiliated Hospital of Qingdao University, Qingdao, 266000 Shandong Province China; 2 Department of Medical Genetics, Affiliated Hospital of Qingdao University, Qingdao, 266003 Shandong Province China; 3 Department of Obstetrics, Affiliated Hospital of Qingdao University, Qingdao, 266003 Shandong Province China

**Keywords:** Gestational Diabetes Mellitus, Systemic Inflammatory Index, Triglyceride-Glucose Index, Chronic inflammation, Mediation analysis

## Abstract

**Background:**

Gestational Diabetes Mellitus (GDM) is known to pose long-term health risks, but the biological mechanisms are not fully understood. The role of the Triglyceride-Glucose Index (TyG) in mediating these effects requires further exploration.

**Method:**

This study investigated the mediation effect of the TyG index on the relationship between GDM history and the Systemic Inflammatory Index (SII) using retrospective data analysis. Mediation analysis quantified the contribution of the TyG index.

**Result:**

Multivariate regression analysis confirmed that the TyG index mediated approximately 40% of the relationship between a history of GDM and elevated SII, highlighting a significant linkage (β = 0.23, 95% CI: 0.18 to 0.28, *P* < 0.0001 for TyG; β = 28.95, 95% CI: 22.88 to 35.02, *P* < 0.0001 for SII). The TyG index was found to mediate approximately 40% of the effect of GDM history on SII, illustrating a significant biological link. These findings highlight the role of metabolic health in influencing systemic inflammation levels associated with GDM.

**Conclusion:**

The study emphasizes the importance of monitoring and managing metabolic and cardiovascular health in women with a history of GDM to mitigate long-term health risks. Future research should focus on prospective studies and the utilization of biomarkers to fully understand the impact of GDM and optimize intervention strategies.

## Introduction

Gestational Diabetes Mellitus (GDM) is a condition diagnosed during pregnancy that affects approximately 2% to 10% of pregnant women [1, 2]. Although GDM typically resolves after childbirth, its long-term health impacts on both mother and child cannot be overlooked [3]. Studies [4] have shown that women with a history of GDM are significantly more likely to develop Type 2 Diabetes Mellitus (T2DM) later in life. Additionally, these women are at a higher risk of other metabolic disorders, such as hypertension and cardiovascular disease [5, 6]. The influence of GDM extends beyond blood sugar control issues. Its direct connection with metabolic health makes GDM a critical window for studying chronic inflammatory states. Inflammation plays a key role in the development of many chronic diseases, including cardiovascular diseases (CVD), metabolic syndrome, and T2DM [7]. Inflammatory markers such as C-reactive protein (CRP) and white blood cell count are often elevated in patients with GDM, indicating that GDM may increase systemic inflammation levels [8, 9].

The Systemic Inflammatory Index (SII), derived from the counts of neutrophils, lymphocytes, and platelets in peripheral blood, has recently been used to assess the state of inflammation within the body [10]. SII has demonstrated potential value in predicting cardiovascular events, cancer progression, and other inflammation-related diseases in various studies [11–13]. Additionally, the Triglyceride-Glucose Index (TyG index), a non-invasive, cost-effective tool for assessing insulin resistance, has been studied in women with GDM. Research indicates that the TyG index correlates positively with the risk of developing metabolic diseases in the future [14, 15]. Although primarily used for evaluating metabolic health, the potential mediating role of the TyG index—namely, its capacity to influence systemic inflammation through metabolic status—remains underexplored.

Therefore, this study aims to fill this knowledge gap by exploring the potential mechanisms through which GDM impacts long-term health, particularly how it influences the TyG index and thereby possibly increases the risk of other chronic diseases. This research is crucial for understanding the comprehensive impact of GDM and providing targeted interventions for its patients, bearing significant clinical and public health implications.

## Materials and methods

### The NHANES study population

**Fig. 1:**
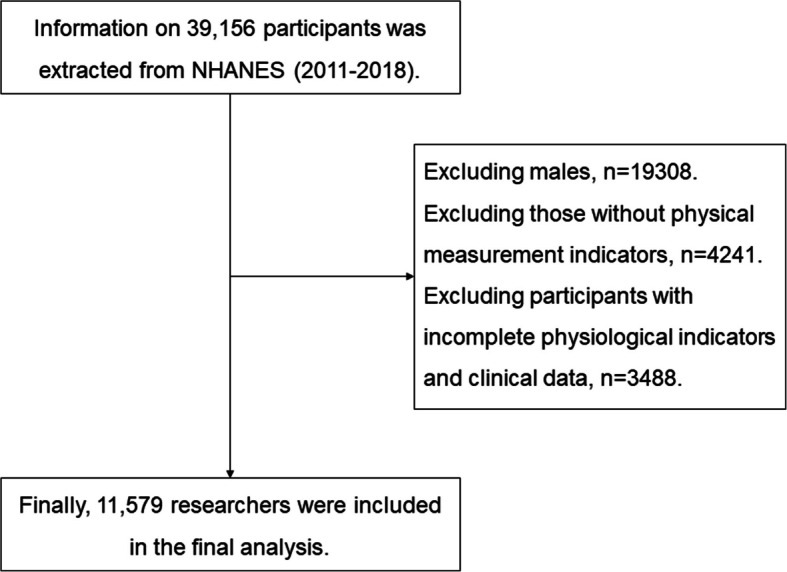
Inclusion and exclusion criteria process

The National Health and Nutrition Examination Survey (NHANES), conducted by the National Center for Health Statistics (NCHS), is an ongoing survey designed to assess the health and nutritional status of the U.S. population. This study utilized data from NHANES for the years 2011-2018. The inclusion criteria encompassed all women over the age of 20 who participated in the NHANES survey during the period from 2011 to 2018. The exclusion criteria were: 1) participants missing key variables such as physical measurements, complete blood counts, lipid profiles, and glucose levels; 2) participants previously diagnosed with malignant tumors or severe chronic diseases, including coronary heart disease, myocardial infarction, stroke, chronic obstructive pulmonary disease, chronic kidney disease, chronic liver disease (such as cirrhosis), and other doctor-diagnosed conditions reported in the NHANES Medical Conditions (MCQ) questionnaire; 3) individuals under 20 years of age. Participants taking medications such as oral hypoglycemic drugs, insulin, lipid-lowering agents, or antihypertensive drugs were not excluded from this study. Menopausal women and those taking hormone replacement therapy (HRT) were also not excluded. As illustrated in Figure 1, after excluding cases that met the above criteria, the final analysis included a sample of 11,579 women.

### GDM medical history, SII assessment, and TyG

Exposure in this analysis involved responses to the reproductive health questionnaire item RHQ162, which asked participants: “Have you ever been told by a doctor or other health professional that you had diabetes during pregnancy? ”. Thus, a history of GDM was determined by self-reported physician diagnosis rather than by applying specific diagnostic criteria. Other pregnancy complications (e.g., gestational hypertension, preeclampsia) were not systematically recorded in the NHANES dataset, and therefore were not included as part of the inclusion or exclusion criteria. Because NHANES is a cross-sectional survey, the interval between the index pregnancy complicated by GDM and the examination date was not available, and thus no specific follow-up range could be determined.

The outcome of this study was the SII. Using an automated hematology analyzer (CoulterDxH 800), lymphocyte, neutrophil, and platelet counts were measured and reported in 10^3 cells/mL. As an inflammation marker, the SII level was determined by multiplying the platelet count by the ratio of neutrophil count to lymphocyte count.

The TyG index, which quantifies insulin resistance, was calculated by combining fasting blood glucose with triglyceride levels. Participants provided blood samples for baseline measurements of fasting serum glucose (FSG) and triglycerides. The calculation formula is as follows:

$$\text{TyG}=\text{ln}\left[\text{Triglycerides }\left(\text{mg}/\text{dL}\right)\times \text{Glucose }\left(\text{mg}/\text{dL }\right)/2\right]$$

### Covariants of NHANES

NHANES employs a complex sampling design and constructs sample weights to produce nationally representative data. These sampling weights are crucial for producing accurate population estimates as each sampled individual does not have an equal probability of being selected. Survey weights for each 2-year cycle are calculated considering factors such as non-response, over-sampling, post-stratification, and sampling error. Further details on sampling weight can be found at NHANES Sample Weight Tutorial. Covariates in NHANES include age (in years), ethnicity (Mexican American, other Hispanic, non-Hispanic White, non-Hispanic Black, and other ethnic groups), marital status (married, other), income-to-poverty ratio (PIR), smoking status (yes or no), alcohol consumption (yes or no), height (cm), weight (kg), Body Mass Index (BMI), waist circumference (cm), and total cholesterol (TC, mg/dL).

### Statistical analysis methods

The NHANES database provides weights for each sample, such as the Mobile Examination Center (MEC) exam weights. Continuous variables are presented as mean ± standard deviation (SD), and categorical variables are presented as proportions. Baseline characteristics of participants were assessed using linear regression models (for continuous variables) or weighted chi-square tests (for categorical variables). Two linear regression models were employed to explore the relationship between a history of GDM and SII. In Model 1, no covariates were adjusted, while in Model 2, covariates such as age, race/ethnicity, marital status, family income to poverty ratio, smoking history, and drinking history were adjusted. Variance inflation factors were calculated, and Spearman's correlation analysis was used to check for collinearity among variables. Beta values (*β*) and 95% confidence intervals (95% CI) were reported. Generalized additive models (GAM) were applied to determine if the relationship between GDM history and SII was non-linear. Subgroup analyses were conducted to evaluate the robustness of the results, and interaction tests were performed. Participants were categorized into different groups based on race/ethnicity, marital status, smoking history, and drinking history, and these stratification factors were also considered as pre-specified potential effect modifiers. Appropriate NHANES sampling weights were used in the statistical analyses. The significance level was set at *p*<0.05.

To assess the mediating role of the TyG index in the association between GDM history and SII, a causal mediation analysis was conducted. We used the 'mediation' R package to quantify direct effects, mediating effects, and total effects. The mediator must demonstrate associations with both the exposure and the outcome [16]. In our study, the independent variable, outcome variable, and mediating variable were represented by GDM history, SII, and TyG, respectively. This approach aligns with the causal mediation analysis framework, facilitating the dissection of the comprehensive impact of GDM history into direct effects on SII and mediated effects via TyG [17]. The PROCESS procedure facilitated mediation analysis, employing 5,000 bootstrap resamples and adjustments reflecting Model III's resampling and adjustments. Data were analyzed using R version 4.2.2 (http://www.r-project.org, The R Foundation). A significance threshold of *P* < 0.05 guided the determination of statistical significance.

## Results

### Baseline characteristics and univariate analysis of the study population

This study recruited 11,579 female participants with an average age of 51.98 ± 16.57 years and an average BMI of 29.86 ± 7.21 kg/m^2^. Among these women, 7.82% had a history of GDM. The participants' average BMI was 29.86 ± 7.21 kg/m^2^, waist circumference was 98.71 ± 16.03 cm, and average TyG index was 8.68 ± 0.69. In the univariate analysis, women with a history of GDM showed significantly higher SII levels (β = 17.03, 95% CI: 1.72 to 32.35, *P* = 0.0293) and a significantly higher TyG index (β = 31.18, 95% CI: 25.23 to 37.12, *P* < 0.0001) compared to those without a history of GDM. A comprehensive overview of baseline characteristics and univariate analysis can be found in Table 1.

**Table 1:** Baseline characteristics and univariate analysis of the study population

	Mean+SD/N(%)	*β* (95% *CI*) *P*-value
Age, years	51.98 ± 16.57	1.12 (0.87, 1.37) <0.0001
Race/Hispanic origin, n%		
Mexican American	1876 (16.20%)	Reference
Other Hispanic	1373 (11.86%)	−39.87 (−55.26, −24.48) <0.0001
Non-Hispanic White	4701 (40.60%)	−19.71 (−31.54, −7.88) 0.0011
Non-Hispanic Black	2472 (21.35%)	43.27 (30.00, 56.54) <0.0001
Other Race - Including Multi-Racial	1157 (9.99%)	−128.46 (−144.66, −112.26) <0.0001
Marital status, n%		
Married	5870 (50.70%)	Reference
Other	5709 (49.30%)	30.49 (22.28, 38.70) <0.0001
History of drinking, n%		
Yes	7371 (63.71%)	Reference
No	4199 (36.29%)	10.17 (1.62, 18.73) 0.0198
Smoking history, n%		
Yes	4330 (37.41%)	Reference
No	7243 (62.59%)	−29.22 (−37.70, −20.73) <0.0001
History of GDM, n%		
No	10673 (92.18%)	Reference
Yes	906 (7.82%)	17.03 (1.72, 32.35) 0.0293
PIR	2.40 ± 1.60	−17.30 (−19.98, −14.61) <0.0001
Weight, kg	76.71 ± 19.93	10.21 (10.12, 10.29) <0.0001
Height, cm	160.12 ± 7.15	3.91 (3.33, 4.48) <0.0001
BMI, kg/cm^2^	29.86 ± 7.21	28.58 (28.35, 28.81) <0.0001
Waist Circumference, cm	98.71 ± 16.03	14.04 (14.02, 14.06) <0.0001
TYG	8.68 ± 0.69	31.18 (25.23, 37.12) <0.0001
TC, mg/dL	197.37 ± 41.23	0.09 (−0.01, 0.19) 0.0670
Fasting glucose, mg/dL	102.23 ± 39.25	0.20 (0.10, 0.31) 0.0002
TG, mg/dL	142.30 ± 95.38	0.17 (0.13, 0.22) <0.0001
WBC, 1000 cells/uL	7.18 ± 2.18	42.54 (40.81, 44.26) <0.0001
Lymphocyte number, 1000 cells/uL	2.25 ± 0.98	−48.49 (−52.61, −44.37) <0.0001
Segmented neutrophils number, 1000 cell/uL	4.16 ± 1.57	96.44 (94.49, 98.38) <0.0001
Platelet count, 1000 cells/uL	253.81 ± 63.56	1.70 (1.64, 1.75) <0.0001

### Multivariate regression and stratified analysis of the association between history of gestational diabetes mellitus and systemic inflammatory index

**Fig. 2:**
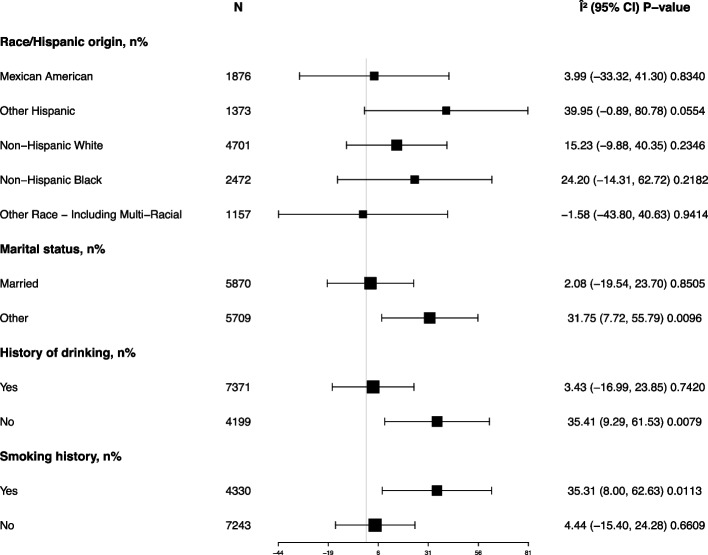
Perform stratified analysis on the SII of the respondents. Shows a stratified analysis of the SII of respondents based on race, marital status, smoking and drinking history, after adjusting for age and household income to poverty ratio

As detailed in Table 2, the multivariate regression analysis explored the relationship between a history of GDM and SII. In Model II, which adjusted for socioeconomic status (PIR) and lifestyle factors (smoking history and drinking history) in addition to age, race, and marital status, the impact of a GDM history on SII remained significant (β = 16.94, 95% CI: 1.12 to 32.77, *P* = 0.0359). This positive correlation suggests that the effect of GDM history is more robust after controlling for these confounding factors. Stratified analysis results indicate that while the positive correlation between GDM history and SII remained consistent across all subgroups (Figure 2), it was particularly significant among those who were not married, non-drinkers, and smokers (all *P* < 0.05).

**Table 2:** Multivariate regression analysis of SII

Outcome: SII	Model I *β* (95% *CI*) *P*-value	Model II *β* (95% *CI*) *P*-value
History of GDM, n%		
No	Reference	Reference
Yes	17.03 (1.72, 32.35) 0.0293	16.94 (1.12, 32.77) 0.0359

Model I no adjusted

Model II adjusted for age(smooth), race/ethnicity, marital status, ratio of family income to poverty(smooth), smoking history and history of drinking

### Mediation analysis of the relationship between history of gestational diabetes mellitus and systemic inflammatory index

**Fig. 3:**
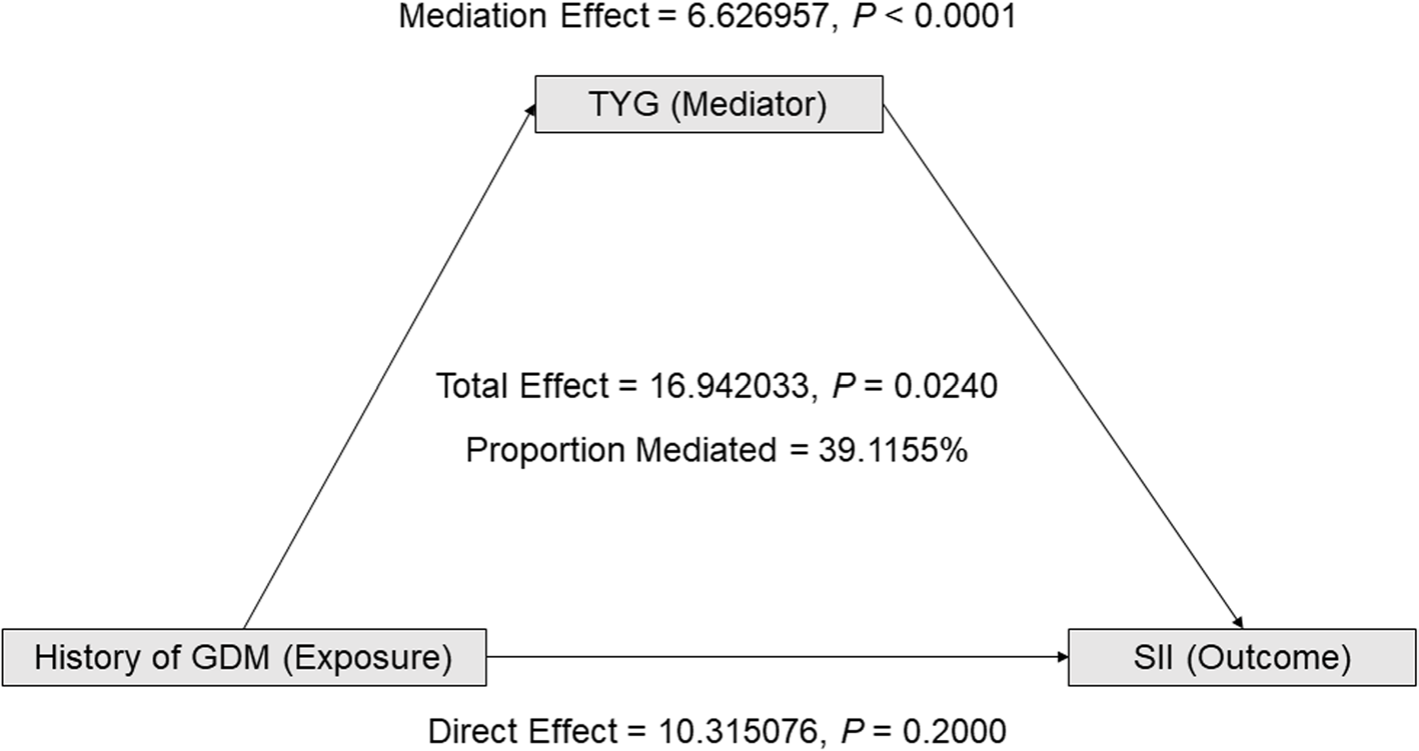
The impact of TyG on the relationship between GDM history and SII. Shows the effect of TyG on the relationship between GDM history and SII. TyG is the mediating factor, GDM history is the exposure factor, and SII is the outcome factor

According to the data presented in Table 3 and Figure 3, there is a significant positive correlation between the history of GDM and the TyG index. This relationship remains significant in Model II, which adjusted for age, race, marital status, socioeconomic status (PIR), and lifestyle factors (smoking and drinking history) (β = 0.23, 95% CI: 0.18 to 0.28, *P* < 0.0001). Additionally, the TyG index is positively correlated with the SII, and the adjusted model shows similar significant results (β = 28.95, 95% CI: 22.88 to 35.02, *P* < 0.0001). The mediation analysis aimed to explore the impact of GDM history on SII revealed that the total effect of GDM history on SII was 16.942033 with a *P* value of 0.0240, indicating a significant positive correlation between GDM history and SII. The direct effect analysis showed that the direct impact of GDM history on SII was 10.315076, but with a *P* value of 0.2000, indicating that this direct impact is not statistically significant. However, it still suggests that GDM history might directly affect SII. Further mediation effect analysis revealed that the mediating effect of the TyG index was 6.626957, with a *P* value less than 0.0001, clearly demonstrating a significant mediating role of TyG between GDM history and SII, accounting for 39.1155% of the impact of GDM history on SII. These Based on current findings, future research should further exploreresults highlight the potential importance of improving metabolic health in reducing chronic inflammation risk among individuals with a history of GDM.

**Table 3:** Multiple regression analysis results of the relationship between SII and TYG, TYG, and GDM

	Model I *β* (95% *CI*) *P*-value	Model II *β* (95% *CI*) *P*-value
Outcome: TYG		
History of GDM, n%		
No	Reference	Reference
Yes	0.24 (0.20, 0.29) <0.0001	0.23 (0.18, 0.28) <0.0001
Outcome: SII	31.18 (25.23, 37.12) <0.0001	28.95 (22.88, 35.02) <0.0001

Model I no adjusted

Model II adjusted for age(smooth), race/ethnicity, marital status, ratio of family income to poverty(smooth), smoking history and history of drinking

## Discussion

The principal finding of this study is that the TyG index plays a significant mediating role in the relationship between a history of GDM and the SII. Through mediation analysis, we found that the TyG index mediates nearly 40% (39.1155%) of the effect of GDM history on SII. These results not only highlight the importance of the TyG index as a bridge between metabolism and inflammation but also emphasize the central role of metabolic health in the development of chronic inflammatory states. This new perspective enhances our understanding of the long-term consequences of GDM, especially regarding the interplay between metabolic status and inflammation.

Compared to existing research, our findings support previous notions that individuals with GDM are at increased risk of developing Type 2 Diabetes Mellitus (T2DM) and cardiovascular diseases postpartum. The existing literature widely acknowledges that individuals with GDM face higher risks of metabolic syndrome and cardiovascular diseases postpartum [18–20], as confirmed by numerous prospective and retrospective studies. For example, several large cohort studies [21, 22] have found that the risk of developing T2DM postpartum in patients with GDM is two to four times that of women without GDM. However, by quantifying the mediating role of the TyG index, our study deepens the understanding of the biological mechanisms underlying the health impacts of GDM. Unlike the existing literature that typically focuses on metabolic status post-GDM, our work highlights the direct link between metabolic status and systemic inflammation and quantifies this connection. For instance, studies have shown that the TyG index, a simple and cost-effective measure of insulin resistance, is not only related to metabolic health but also closely linked to systemic inflammation levels [23]. This approach provides a more comprehensive framework for understanding the complex interactions between GDM and long-term health risks.

The history of GDM increases SII by elevating the TyG index, possibly because increased insulin resistance leads to higher production of inflammatory markers. The rise in the TyG index reflects intensified insulin resistance, which is associated with increased inflammatory activity. Insulin resistance is considered a key driver of chronic inflammation and can influence the inflammatory state through various biological pathways. For example, insulin resistance can lead to increased infiltration of inflammatory cells in adipose tissue, which release pro-inflammatory cytokines such as tumor necrosis factor-alpha and interleukin-6, further promoting inflammation. Additionally, insulin resistance is associated with endothelial dysfunction, which may exacerbate the systemic inflammatory environment by affecting vascular inflammatory responses. This inflammation exacerbated by insulin resistance could intensify systemic inflammation through several mechanisms, including increased oxidative stress. Oxidative stress, caused by an overproduction of free radicals and an imbalance in antioxidant defenses, can directly damage cellular components such as lipids, proteins, and DNA, and activate multiple inflammatory signaling pathways [24–26]. This process not only promotes local inflammatory responses but could also impact overall health through the release of circulating inflammatory mediators.

Despite the valuable insights provided, this study has several limitations. First, the cross-sectional design of NHANES restricts our ability to establish temporal or causal relationships between a history of GDM, systemic inflammation, and metabolic markers, and the reliance on self-reported GDM history may introduce recall bias. Longitudinal cohort studies with biomarker verification of GDM and metabolic health are needed to confirm these associations. Second, medication use (e.g., glucose-lowering, lipid-lowering, or antihypertensive drugs) may influence TyG and systemic inflammation levels, but was not specifically adjusted in our analyses, which should be acknowledged as a limitation. Third, menopausal status and the use of HRT may influence metabolic and inflammatory markers, but were not specifically adjusted in our analyses, which should be acknowledged as a limitation. Although we adjusted for socioeconomic status (PIR) and lifestyle factors (smoking and drinking history) as well as demographic covariates, other potential confounders such as dietary habits, physical activity, and comorbid health conditions were not available in NHANES and could not be fully accounted for. This may have influenced the robustness of our findings. Fourth, although SII has been reported to be associated with CVD and T2DM, these outcomes were not assessed in our analysis. Future studies should evaluate whether SII can predict the development of CVD and T2DM among women with a history of GDM. Fifth, other pregnancy complications such as gestational hypertension and preeclampsia, which may also affect metabolic and inflammatory markers, were not available in the NHANES dataset and therefore could not be considered in this analysis. Sixth, because NHANES does not capture the interval since the index pregnancy, heterogeneity in the timing of outcome assessment may exist, potentially influencing the observed associations. Future longitudinal studies with detailed follow-up data are needed to clarify the temporal dynamics between GDM history, metabolic status, and systemic inflammation.

Based on current findings, future research should further explore the impact of GDM history on other inflammation-related diseases, such as autoimmune diseases and chronic kidney disease. In addition, validation in external cohorts with broader demographic and geographic diversity is necessary to confirm the generalizability of these findings. Studies should consider assessing the metabolic and inflammatory statuses of women with gestational diabetes across different racial and economic backgrounds to better understand how these variables affect health outcomes post-GDM. Finally, research into how to improve metabolic health and reduce the long-term risk of inflammation through intervention measures is also a crucial direction for future studies. While our findings suggest that TyG may mediate the association between GDM history and systemic inflammation, these conclusions should be interpreted with caution given the cross-sectional design and unmeasured confounders. Prospective studies with detailed follow-up and validation in external populations are needed to confirm the robustness and generalizability of these associations.

## Conclusion

This study confirmed that a history of GDM significantly increases the SII through the elevation of the TyG, revealing complex biological links between GDM and long-term health issues. The TyG index accounted for approximately 40% of the mediating effect on the impact of GDM history on SII, highlighting the potential importance of improving metabolic health to reduce the risk of chronic inflammation. These findings support the importance of enhancing monitoring of metabolic and cardiovascular health in women with a history of GDM to prevent and manage potential long-term health consequences. Future research should explore the comprehensive impact of GDM on women's health through prospective designs and the use of biomarkers, thus optimizing intervention strategies.

## Data Availability

No datasets were generated or analysed during the current study.

## References

[1] Zhang C, Catalano P. Screening for gestational diabetes. JAMA. 2021;326(6):487–9. 10.1001/jama.2021.12190. 34374733. 34374733 10.1001/jama.2021.12190

[2] Sweeting A, Hannah W, Backman H, Catalano P, Feghali M, Herman WH, et al. Epidemiology and management of gestational diabetes. Lancet. 2024;404(10448):175–92. 10.1016/S0140-6736(24)00825-0. 38909620 10.1016/S0140-6736(24)00825-0

[3] Sweeting A, Wong J, Murphy HR, Ross GP. A clinical update on gestational diabetes mellitus. Endocr Rev. 2022;43(5):763–93. 10.1210/endrev/bnac003. 35041752 10.1210/endrev/bnac003 PMC9512153

[4] Malaza N, Masete M, Adam S, Dias S, Nyawo T, Pheiffer C. A systematic review to compare adverse pregnancy outcomes in women with pregestational diabetes and gestational diabetes. Int J Environ Res Public Health. 2022;19(17):10846. 10.3390/ijerph191710846. 36078559 10.3390/ijerph191710846 PMC9517767

[5] Ramlakhan KP, Johnson MR, Roos-Hesselink JW. Pregnancy and cardiovascular disease. Nat Rev Cardiol. 2020;17(11):718–31. 10.1038/s41569-020-0390-z. 32518358 10.1038/s41569-020-0390-z

[6] Bianco ME, Josefson JL. Hyperglycemia during pregnancy and long-term offspring outcomes. Curr Diab Rep. 2019;19(12):143. 10.1007/s11892-019-1267-6. 31754898 10.1007/s11892-019-1267-6 PMC7008468

[7] Dinarello CA. Interleukin-1 in the pathogenesis and treatment of inflammatory diseases. Blood. 2011;117(14):3720–32. 10.1182/blood-2010-07-273417. 21304099 10.1182/blood-2010-07-273417 PMC3083294

[8] Jamilian M, Mirhosseini N, Eslahi M, Bahmani F, Shokrpour M, Chamani M, et al. The effects of magnesium-zinc-calcium-vitamin D co-supplementation on biomarkers of inflammation, oxidative stress and pregnancy outcomes in gestational diabetes. BMC Pregnancy Childbirth. 2019;19(1):107. 10.1186/s12884-019-2258-y. 30922259 10.1186/s12884-019-2258-y PMC6440090

[9] Huang S, Chen J, Cui Z, Ma K, Wu D, Luo J, et al. Lachnospiraceae-derived butyrate mediates protection of high fermentable fiber against placental inflammation in gestational diabetes mellitus. Sci Adv. 2023;9(44):eadi7337. 10.1126/sciadv.adi7337. 37922350 10.1126/sciadv.adi7337 PMC10624355

[10] Mahemuti N, Jing X, Zhang N, Liu C, Li C, Cui Z, et al. Association between systemic immunity-inflammation index and hyperlipidemia: a population-based study from the NHANES (2015–2020). Nutrients. 2023;15(5):1177. 10.3390/nu15051177. 36904176 10.3390/nu15051177 PMC10004774

[11] Liu B, Wang J, Li YY, Li KP, Zhang Q. The association between systemic immune-inflammation index and rheumatoid arthritis: evidence from NHANES 1999–2018. Arthritis Res Ther. 2023;25(1):34. 10.1186/s13075-023-03018-6. 36871051 10.1186/s13075-023-03018-6 PMC9985219

[12] Tang Y, Peng B, Liu J, Liu Z, Xia Y, Geng B. Systemic immune-inflammation index and bone mineral density in postmenopausal women: a cross-sectional study of the national health and nutrition examination survey (NHANES) 2007–2018. Front Immunol. 2022;13:975400. 10.3389/fimmu.2022.975400. 36159805 10.3389/fimmu.2022.975400 PMC9493473

[13] Cao Y, Li P, Zhang Y, Qiu M, Li J, Ma S, et al. Association of systemic immune inflammatory index with all-cause and cause-specific mortality in hypertensive individuals: results from NHANES. Front Immunol. 2023;14:1087345. 10.3389/fimmu.2023.1087345. 36817427 10.3389/fimmu.2023.1087345 PMC9932782

[14] Alizargar J, Bai CH, Hsieh NC, Wu SV. Use of the triglyceride-glucose index (TyG) in cardiovascular disease patients. Cardiovasc Diabetol. 2020;19(1):8. 10.1186/s12933-019-0982-2. PMID:31941513;PMCID:PMC6963998. 31941513 10.1186/s12933-019-0982-2 PMC6963998

[15] Zeng Y, Yin L, Yin X, Zhao D. Association of triglyceride-glucose index levels with gestational diabetes mellitus in the US pregnant women: a cross-sectional study. Front Endocrinol (Lausanne). 2023;14:1241372. 10.3389/fendo.2023.1241372. 37881497 10.3389/fendo.2023.1241372 PMC10597685

[16] Tofighi D, MacKinnon DP. RMediation: an R package for mediation analysis confidence intervals. Behav Res Methods. 2011;43(3):692–700. 10.3758/s13428-011-0076-x. PMID:21487904;PMCID:PMC3233842. 21487904 10.3758/s13428-011-0076-x PMC3233842

[17] Valeri L, Vanderweele TJ. Mediation analysis allowing for exposure-mediator interactions and causal interpretation: theoretical assumptions and implementation with SAS and SPSS macros. Psychol Methods. 2013;18(2):137–50. 10.1037/a0031034. Epub 2013 Feb 4. Erratum in: Psychol Methods. 2013 Dec;18(4):474. PMID: 23379553; PMCID: PMC3659198. 23379553 10.1037/a0031034 PMC3659198

[18] Kramer CK, Campbell S, Retnakaran R. Gestational diabetes and the risk of cardiovascular disease in women: a systematic review and meta-analysis. Diabetologia. 2019;62(6):905–14. 10.1007/s00125-019-4840-2. 30843102 10.1007/s00125-019-4840-2

[19] Rayes B, Ardissino M, Slob EAW, Patel KHK, Girling J, Ng FS. Association of hypertensive disorders of pregnancy with future cardiovascular disease. JAMA Netw Open. 2023;6(2):e230034. 10.1001/jamanetworkopen.2023.0034. 36800181 10.1001/jamanetworkopen.2023.0034 PMC9938428

[20] Choudhury AA, Devi Rajeswari V. Gestational diabetes mellitus - a metabolic and reproductive disorder. Biomed Pharmacother. 2021;143:112183. 10.1016/j.biopha.2021.112183. 2021 Sep 21. PMID: 34560536. 34560536 10.1016/j.biopha.2021.112183

[21] Zhu Y, Zhang C. Prevalence of gestational diabetes and risk of progression to type 2 diabetes: a global perspective. Curr Diab Rep. 2016;16(1):7. 10.1007/s11892-015-0699-x. 26742932 10.1007/s11892-015-0699-x PMC6675405

[22] Wicklow B, Retnakaran R. Gestational diabetes mellitus and its implications across the life span. Diabetes Metab J. 2023;47(3):333–44. 10.4093/dmj.2022.0348. 36750271 10.4093/dmj.2022.0348 PMC10244196

[23] Huo RR, Liao Q, Zhai L, You XM, Zuo YL. Interacting and joint effects of triglyceride-glucose index (TyG) and body mass index on stroke risk and the mediating role of TyG in middle-aged and older Chinese adults: a nationwide prospective cohort study. Cardiovasc Diabetol. 2024;23(1):30. 10.1186/s12933-024-02122-4. 38218819 10.1186/s12933-024-02122-4 PMC10790273

[24] Yang T, Li G, Wang C, Xu G, Li Q, Yang Y, et al. Insulin resistance and coronary inflammation in patients with coronary artery disease: a cross-sectional study. Cardiovasc Diabetol. 2024;23(1):79. 10.1186/s12933-024-02159-5. 38402392 10.1186/s12933-024-02159-5 PMC10893710

[25] Olefsky JM, Glass CK. Macrophages, inflammation, and insulin resistance. Annu Rev Physiol. 2010;72:219–46. 10.1146/annurev-physiol-021909-135846. PMID: 20148674. 20148674 10.1146/annurev-physiol-021909-135846

[26] Shoelson SE, Lee J, Goldfine AB. Inflammation and insulin resistance. J Clin Invest. 2006;116(7):1793–801. 10.1172/JCI29069. Erratum.In:JClinInvest.2006Aug;116(8):2308.PMID:16823477;PMCID:PMC1483173. 16823477 10.1172/JCI29069 PMC1483173

